# Emmenagogue Medicines

**Published:** 1843-10

**Authors:** 


*Emmenagogue Medicines.*—Of emmenagogue remedies, or those medicines that are supposed to possess a specific power over the womb, and which have been resorted to by medical men in cases of amenorrhœa for reproducing the menstrual secretion in conjunction with the general treatment, the folowing appear to have proved most successful.

Savine—from five to ten grains of the powdered leaves three times a day, or a drachm of the compound tincture twice a day.

Dr. Locock recommends a combination of myrrh, aloes, sulphate of iron; and the essential oil of savine.

Ergot of rye—five to ten grains of the powder two or three times a day, boiled in a little milk. In very irritable habits the ergot must be cautiously administered, as it has been found, after a few days, to produce sometimes violent and even highly dangerous spasmodic attacks.

Iodine in form of tincture, with hydriodate of potash—ten to twenty or thirty drops, two or three times a day.

Strychine—one-tenth to one-fourth of a grain, two or three times a day. This medicine must be suspended for a few days, should headache or twitching of the muscles follow its exhibition.

Madder, myrrh, guaiacum, mustard-seed, valerian, electricity, aloes, have all had their advocates.

In Dr. Ashwell's report of obstetric cases, vol. iii., Guy's Hospital Reports, out of nine cases of amenorrhœa admitted into the hospital, seven simple and two complicated, "six of the simple cases occurred in the persons of delicate females, and the remaining patient was a strong, active, plethoric girl of nineteen. The former was treated by aperients and metallic tonics; four employed the ammoniacal injection with benefit; while electricity in some cases seemed to be of great service in exciting menstruation."

In cases of *amenorrhœa combined with epilepsy*, Dr. A. employed the following formula, recommended by Dr. Bright:—

℞ Pulv. digitalis, gr. j.; Pulv. myrrhæ, gr. ij.; Ferri sulphatis, gr. j.; Syr. q. s. ft. pil. ter die cap.

"Under this treatment the fits became diminished in number, and the menstrual function normally established."

*Braithwaite's Retrospect.*

